# Integration of SBAS-InSAR and RFE-RF-XGBoost for Landslide Vulnerability Assessment: A Case Study in Zhaotong City, Yongshan County

**DOI:** 10.3390/s25237215

**Published:** 2025-11-26

**Authors:** Junjie Huang, Mengyao Shi, Yuyin Ma, Cheng Huang, Weiheng Qian, Fuxiang Sun, Xiaoqing Zuo

**Affiliations:** 1 Faculty of Land and Resources Engineering, Kunming University of Science and Technology, Kunming 650093, China; 20242201097@kust.edu.cn (J.H.); qianweiheng@stu.kust.edu.cn (W.Q.); sun@stu.kust.edu.cn (F.S.); zxg@kust.edu.cn (X.Z.); 2 Yunnan Key Laboratory of Intelligent Monitoring and Spatiotemporal Big Data Governance of Natural Resources, Kunming University of Science and Technology, Kunming 650093, China; 3 Kunming Engineering Investigation Company, Kunming 650000, China; m13577009026@163.com; 4 Yunnan Institute of Geo-Environment Monitoring, Kunming 650216, China; hch2377@stu.kust.edu.cn

**Keywords:** SBAS-InSAR, landslide susceptibility assessment, RFE-RF-XGBoost, machine learning, Yongshan County

## Abstract

Yongshan County in northeastern Yunnan Province is a frequent geological hazard zone. Based on previous detailed geological hazard surveys, the county contains 455 landslide hazard sites, primarily distributed in the western and northern regions. Influenced by multiple factors including rainfall, earthquakes, human activities, and reservoir water storage, it is challenging to evaluate their development using a single indicator. Therefore, there is an urgent need to conduct landslide susceptibility assessments that integrate deformation rate characteristics. However, existing studies in this region have only considered static spatial factors such as slope aspect, elevation, and lithology. Traditional landslide susceptibility assessments often struggle to balance zoning accuracy with timeliness, leading to biased results and limited update efficiency. This study employs SBAS-InSAR technology to capture surface deformation rates and utilizes machine learning models to partition landslide susceptibility distribution maps. It innovatively introduces an RFE-RF-XGBoost model to reduce partitioning errors and enhance the accuracy of landslide susceptibility mapping. Experiments utilized 147 Sentinel-1A and 14 LT-1 scenes. Through five-fold cross-validation, 13 influencing factors were selected. The RFE-RF-XGBoost model was trained via hyperparameter optimization and compared against four conventional models (CatBoost, LightGBM, XGBoost, RF). After validating the predictive performance of different models via ROC curves, the prediction results at each level were analyzed using Accuracy, Precision, Recall, and F1 metrics. Results indicate that all five machine learning models demonstrate effective zoning capabilities. Among them, the RFE-RF-XGBoost model achieves optimal mapping performance. Compared to the other four models, it reduces the proportion of low-risk zones by 2–4% while increasing the proportion of extremely high-risk zones by approximately 2–12%, with an AUC value reaching around 0.95. Field investigations further validated that this approach enhances landslide interpretation accuracy by integrating SBAS-InSAR technology with remote sensing techniques.

## 1. Introduction

Landslides rank among the world’s most prevalent geological hazards. In northeastern Yunnan Province, China, frequent landslides pose severe threats to the lives and property of local residents. These failures result from fragile geological conditions, seasonal rainfall, seismic activity, urban development, and large-scale reservoir impoundment [1]. Reliable landslide susceptibility mapping is crucial for identifying potential landslide zones and managing associated risks [2,3].

Landslide susceptibility quantifies the spatial probability of landslide occurrence. Generally, the greater the similarity to known landslide geological environments, the higher the likelihood of landslide occurrence [4,5,6]. With the advancement of computer networks, machine learning models have gradually replaced traditional statistical models, becoming a research hotspot in landslide susceptibility mapping [7,8]. Widely used models include artificial neural network models [9,10] and Bayesian network models [11]. However, a single machine learning model rarely fits all scenarios [12]. Ensemble modeling, regarded as a frontier topic in Earth sciences, constructs learning models using a set of classifiers to efficiently handle complex multidimensional data [13,14]. For instance, Huang et al. [4,15,16] employed bagging-based ensemble random forests for landslide susceptibility assessment; Liu Haizhi et al. [14] applied ensemble learning to identify landslide-prone areas in mountainous small watersheds; T. Zhang et al. [17] integrated bagging with four benchmark models—optimal first decision trees, functional trees, support vector machines, and classification and regression trees—to achieve superior prediction performance. Nevertheless, no globally established framework or guidelines exist, and determining optimal modeling strategies remains a key research focus [14].

Previous landslide susceptibility assessments failed to account for dynamic factors, focusing solely on static influencing factors such as slope aspect, elevation, lithology, and distance to rivers [18]. For instance, triggering events can reactivate ancient landslides, while engineering activities may induce new landslides. If such information is not incorporated into updated landslide susceptibility maps, false negative errors will inevitably occur [19].

Therefore, some studies have begun to attempt to introduce InSAR technology into landslide susceptibility assessment. For example, Piacentini et al. [20] utilized PS-InSAR to update landslide inventories for susceptibility mapping; Ciampalini et al. [21] proposed a dynamic assessment method that integrates InSAR deformation with susceptibility maps through empirical matrices; Calvello et al. [22] combined PS-InSAR with susceptibility models to assess the activity status of slow-moving landslides. However, most of these studies used InSAR data for post-processing correction or as independent static layers, failing to deeply integrate it as a core dynamic feature into the machine learning training process. Furthermore, at the model level, how to effectively select features and optimize prediction performance through ensemble learning remains a focus of current research.

In recent years, substantial progress has been made in landslide susceptibility mapping research both domestically and internationally. Although InSAR technology has been applied to landslide studies, most efforts remain confined to simple deformation monitoring or landslide inventory updates, often relying on a single data source (primarily Sentinel-1). To overcome these limitations, this study expands the data framework: in addition to utilizing long-term time-series observations from Sentinel-1A (147 scenes covering May 2022 to March 2025), it incorporates 14 scenes of LT-1 satellite imagery (covering 20 January 2024, to 12 February 2025) as supplementary data.

The present study aims to achieve the following objectives: (1) to construct a landslide susceptibility assessment framework integrating SBAS-InSAR dynamic deformation data with multi-source geo-environmental factors; (2) to propose a novel RFE-RF-XGBoost hybrid ensemble model for enhanced accuracy and reliability in susceptibility zoning. The methodological contribution lies in introducing a hybrid ensemble learning framework where Recursive Feature Elimination (RFE) coupled with Random Forest (RF) enables robust feature selection, subsequently leveraged by XGBoost as a powerful predictor. This design specifically addresses accuracy limitations in conventional assessments caused by feature redundancy and single-model constraints. Compared to individual models or other ensemble techniques, our approach offers dual advantages: effectively eliminating irrelevant and redundant features prior to modeling, thereby improving generalizability and interpretability, while XGBoost’s built-in regularization and gradient boosting mechanisms further reduce variance and bias, yielding more precise zoning in complex geological settings. The study’s scientific novelty and advantages are thus demonstrated through multi-source data fusion, innovative model architecture, and dynamic assessment processes that treat InSAR-derived deformation rates as core input features rather than post-processing tools.

Based on this, our study innovatively integrates time-series deformation rates obtained from SBAS-InSAR as dynamic features, which are input together with static environmental factors into integrated machine learning models for training. This approach aims to achieve an upgrade from “static susceptibility” to “dynamic risk” assessment, thereby enhancing the accuracy and timeliness of landslide identification.Building upon this foundation, an RFE-RF-XGBoost model accounting for boundary conditions was constructed to reduce errors in landslide susceptibility zoning and improve mapping precision. By leveraging complementary multi-source satellite data, this study enhances the temporal continuity and spatial coverage of InSAR deformation monitoring. It improves deformation detection reliability in complex terrain and vegetation-covered areas, achieving deeper coupling and accuracy improvements between InSAR data and susceptibility mapping. Finally, field investigations validate the interpretation accuracy and susceptibility zoning map correctness.

Section 2 systematically introduces the study area overview and sources of multi-source landslide risk factor data. Section 3 focuses on the SBAS-InSAR deformation monitoring methodology and the RFE-RF-XGBoost modeling workflow. Section 4 details InSAR data processing and hazard identification. Section 5 presents experimental studies and results. Section 6 concludes the paper.

## 2. Study Area and Data Sources

### 2.1. Study Area Overview

**Figure 1:**
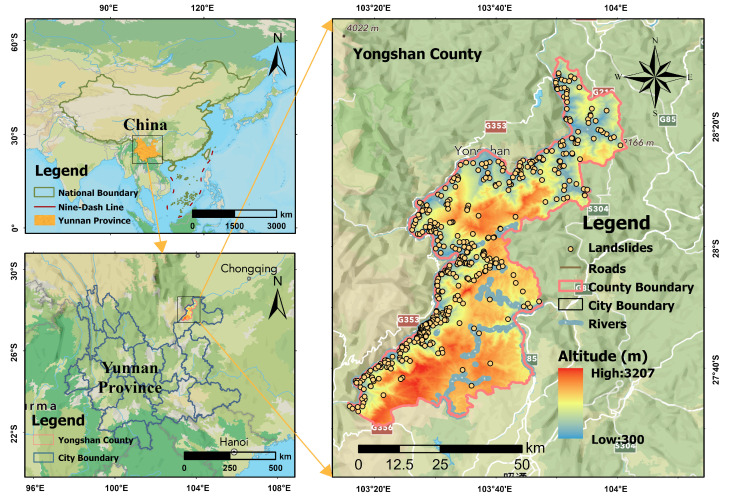
Location of the study area and distribution of landslide points.

Yongshan County is located in Zhaotong City, northeastern Yunnan Province, China (Figure 1). The region features complex topography with elevations ranging from 400 to 3200 m, characterized by deep valleys and steep slopes. Geologically, the area belongs to the Yangtze Paraplatform with well-developed fractures and folds. The stratum lithology is mainly composed of carbonate rocks, clastic rocks, and Quaternary deposits.

The study area experiences a subtropical monsoon climate with an average annual rainfall of 1500–2500 mm, concentrated mainly from May to October. This rainfall pattern significantly influences landslide occurrence. Additionally, the region is affected by seismic activity due to its location in an active tectonic zone. Human engineering activities, including road construction, mining, and urban expansion, have further exacerbated slope instability.

### 2.2. Data Sources

The primary data sources for this study include Sentinel-1A radar data, optical remote sensing data, geological structure and DEM data, as well as vector data for disaster points, roads, and water systems. Detailed data are presented in Table 1.

### 2.3. Landslide Inventory and Landslide Impact Factors

**Figure 2:**
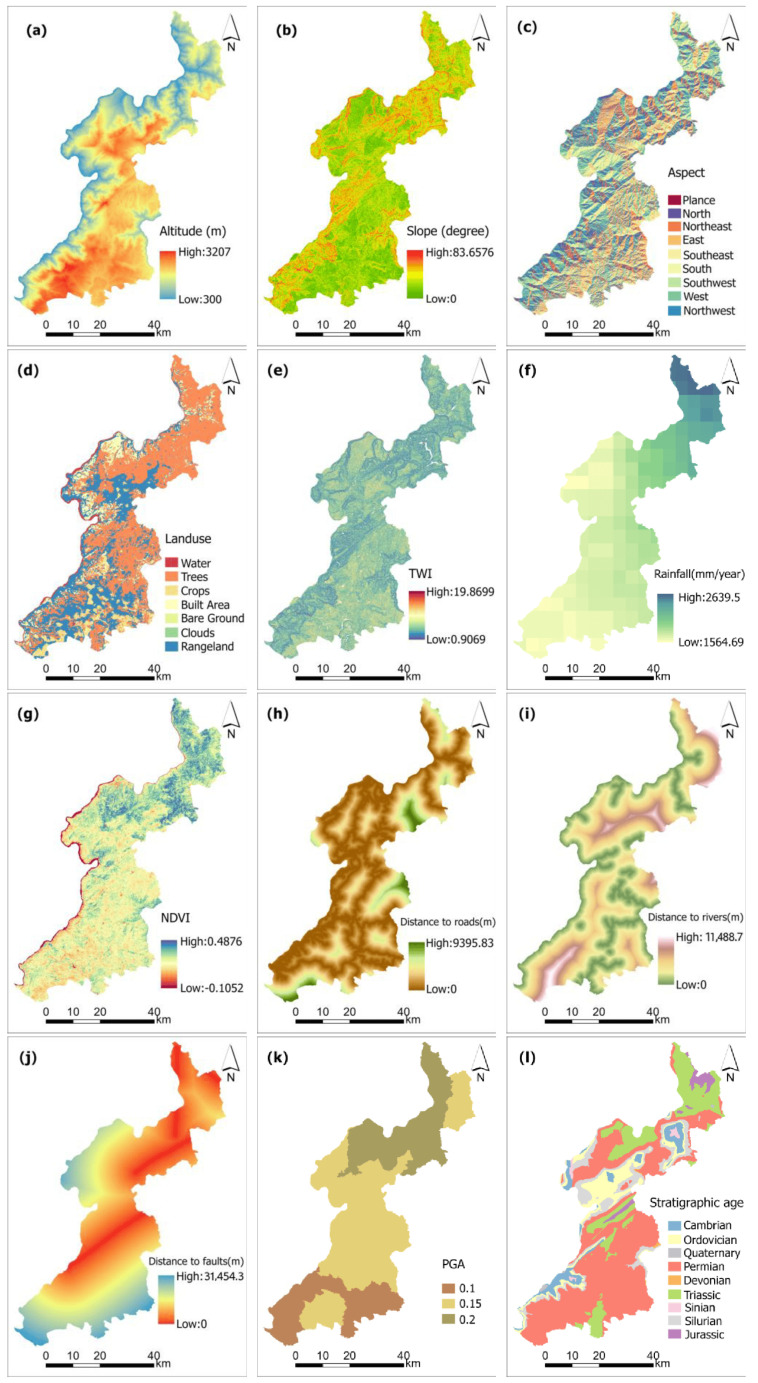
Spatial distribution of landslide evaluation factors used in the susceptibility assessment: (**a**) Altitude; (**b**) Slope; (**c**) Aspect; (**d**) Landuse; (**e**) Terrain Relief; (**f**) TWI; (**g**) Rainfall; (**h**) Distance to faults; (**i**) Distance to rivers; (**j**) NDVI; (**k**) Distance to roads; (**l**) Stratigraphic age.

According to the statistics from the detailed investigation and risk assessment of geological hazards in Yongshan County, Yunnan Province, there are 455 landslide hazard sites within the county. A landslide distribution map for the study area has been constructed (see Figure 2). Most landslides are linearly distributed, primarily along one side of the Jinsha River, due to local fragile geological conditions, seasonal rainfall, earthquakes, urban residential development, and large-scale reservoir impoundment. Additionally, landslides are concentrated within 1 km of roads and along fault zones.

This study categorizes geological hazard impact indicators into five groups: topography and landforms, geological conditions, hydrometeorological factors, human activities, and vegetation ecology. Topographic factors include elevation, slope gradient, aspect, and terrain undulation; geological factors encompass lithology, distance to fault zones, and peak ground acceleration during earthquakes; hydrometeorological factors comprise precipitation, terrain wetness index (TWI), and distance to rivers; human activity factors involve proximity to roads. The normalized difference vegetation index (NDVI) and land use patterns constitute vegetation ecological factors.

Based on field surveys and evaluation accuracy requirements, data for each indicator factor were collected from sources listed in Table 1. The Slope, Aspect, and Relief factors were extracted from elevation data using the Raster Surface tool in ArcGIS Pro 3.0.1; Lithology and stratigraphic age information were extracted from geological maps and structural outline maps. The buffer tool in ArcGIS Pro processed data for fault zones, water systems, roads, etc. Google Earth Engine interpolated 2023 average precipitation data to generate a raster dataset of annual precipitation for Yongshan County, Zhaotong City. NDVI values for Yongshan County, Zhaotong City, at 30-m resolution for 2023 were obtained via Google Earth Engine. After determining the distribution of landslides across the entire region based on remote sensing imagery data, the historical landslide inventory information was updated and supplemented using field surveys and detailed landslide investigation materials.

Using 455 historical landslide sites and 73 interpreted landslide sites within the study area as positive samples, circular buffers with a 1 km radius centered on each positive sample were created in ArcGIS. Rivers were masked outside these buffers. The “Create Random Points” function in ArcGIS generated 528 random points as negative samples to construct the machine learning sample dataset. A total of 70% of the samples were selected as training data, while 30% served as test data. A 30 m × 30 m grid cell was adopted as the basic evaluation unit, ultimately dividing the area into 3,154,444 grid cells. A landslide susceptibility evaluation index system was constructed using 12 factors, including elevation, slope gradient, and slope aspect. Continuous factors such as distance to rivers, roads, and faults were categorized by interval. Discrete factors like stratigraphic age, slope aspect, and land use type were classified based on actual conditions, while the remaining factors were categorized using the natural break method. The classification results for each evaluation factor are shown in Figure 2.

## 3. Methodology

### 3.1. Research Approach

**Figure 3:**
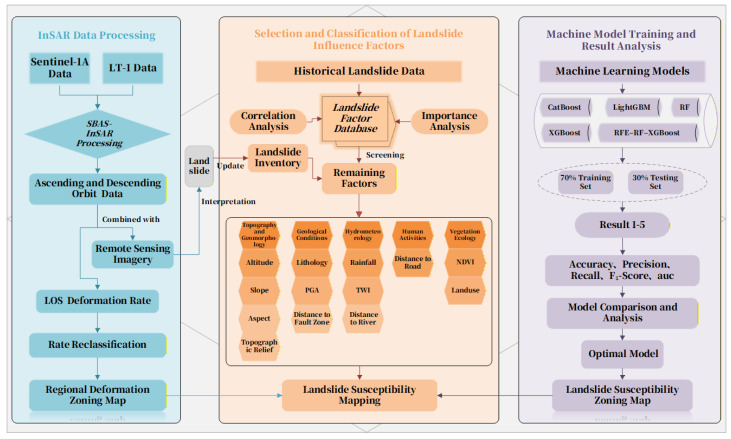
Landslide susceptibility evaluation flowchart showing the integrated approach of SBAS-InSAR and machine learning.

Sentinel-1 SAR data for Yongshan County, covering 5 May 2022 to 26 March 2025, were obtained from the ASF Data Search platform. Then, employ SBAS-InSAR technology to obtain the surface deformation rate and cumulative deformation amount along the Line-of-Sight (LOS) direction for Yongshan County. After reclassifying the rates, generate a regional deformation zoning map. Integrate this with Gaofen satellite imagery and Google Earth images to interpret landslides and potential landslide zones, updating the landslide catalog. Based on the study area’s topography and disaster distribution, thirteen susceptibility evaluation indicators were selected, covering topography, geology, hydrometeorology, human activities, and vegetation ecology. These indicators were classified and standardized. The Pearson correlation coefficient method was applied to test the correlation among the initial indicators, eliminating highly correlated factors to establish a landslide susceptibility evaluation index system. An equal number of landslide and non-landslide points were selected as training data (70% for model training, 30% for testing). To ensure the spatial generalizability of the model and avoid overestimation of model performance due to spatial autocorrelation, this study adopted a spatial block cross-validation strategy [23,24] in the five-fold cross-validation process. The study area was divided into multiple spatially discontinuous blocks to ensure spatial independence between the training and test sets. A landslide susceptibility evaluation model was constructed in a Python environment using the RFE-RF-XGBoost machine learning approach for Yongshan County. Prediction results across all susceptibility levels were analyzed using accuracy, precision, recall, and F1 score to analyze prediction results across different hazard levels. The model’s predictive performance was validated using ROC curves to assess fitting accuracy and predictive capability. A landslide susceptibility hazard map was generated by overlaying the susceptibility classification map with the regional deformation zoning map, completing the final landslide susceptibility mapping (as shown in Figure 3).

### 3.2. RFE-RF-XGBoost Model

**Figure 4:**
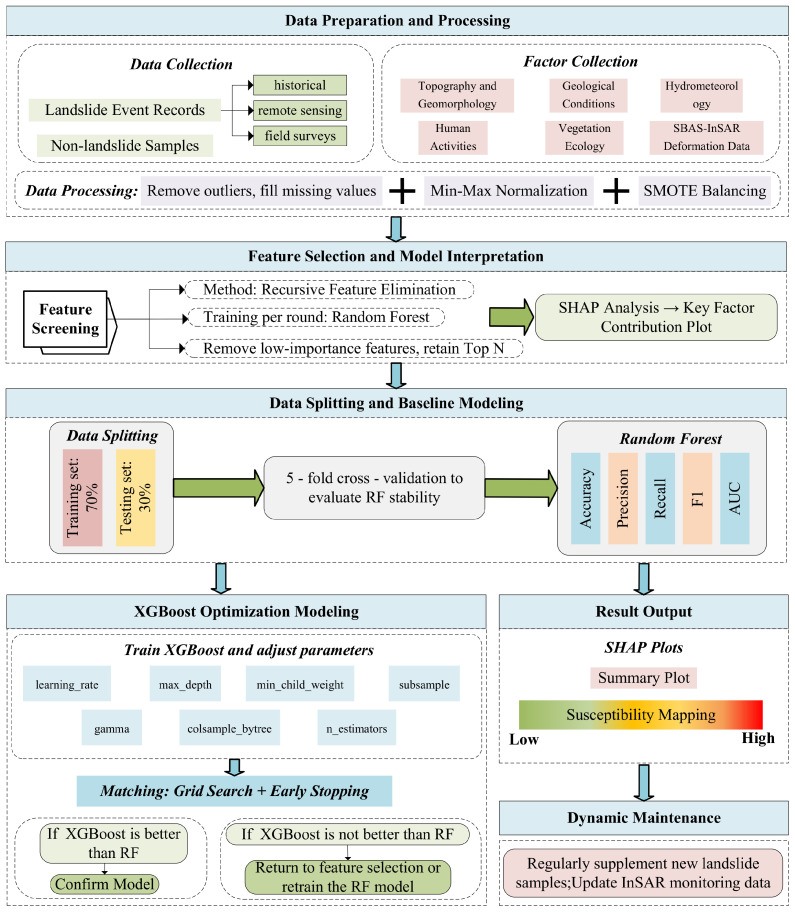
RFE-RF-XGBoost model construction flowchart.

The overall workflow of the RFE-RF-XGBoost model construction is illustrated in Figure 4, which encompasses all steps from data preparation to dynamic maintenance.

(1) Data Preparation and Preprocessing: Collect landslide events and non-landslide samples while gathering factors such as topography, geology, hydrology, precipitation, and land use. Integrate SBAS-InSAR technology, then perform data cleaning, min-max normalization, and SMOTE balancing to enhance the model’s ability to identify rare landslide samples.

(2) Feature Selection: Employ RFE combined with RF for factor screening to obtain the optimal feature subset. Further interpret key factor contributions using SHAP to enhance transparency.

(1) $$S*=RFE_{RF}\left(D\right)$$

Here, *D* represents the original dataset and S* denotes the selected optimal feature subset.

(3) Data Partitioning: Allocate 70% to the training set and 30% to the test set. Utilize 5-fold cross-validation (CV) during training to assess model stability.

(4) Random Forest Modeling: Trained RF models based on optimal feature subset and evaluated model performance (accuracy, precision, recall, F1, AUC, etc.) on the test set as the baseline model.

(5) XGBoost Training: Built XGBoost models using the same feature subset, with the objective function defined as:

(2) $$Obj\left(\theta \right)=\sum_{i=1}^{n}l\left(y_{i},{\hat{y}}_{i}\right)+\sum_{k}\Omega \left(f_{k}\right),\;\Omega \left(f\right)=\gamma T+\frac{1}{2}\lambda {∥w∥}^{2}$$

Here, *l* represents the loss function, Ω denotes the regularization term, $f_{k}$ indicates the *k*-th tree, and θ represents the parameter set. Set hyperparameter tuning ranges: learning_rate, max_depth, min_child_weight, gamma, subsample, colsample_bytree, and n_estimators. Grid search was employed for hyper-parameter tuning, with the search space listed in Table 2.

To guarantee reproducibility, a random seed (random_state = 42) was fixed in all experiments. Early stopping was implemented during training: training is automatically terminated if the validation loss does not decrease for 10 consecutive rounds, which effectively mitigates overfitting. All continuous input features were scaled to the interval [0,1] through Min–Max normalization. The final optimal configuration is: learning_rate = 0.1, max_depth = 7, min_child_weight = 3, gamma = 0.1, subsample = 0.9, colsample_bytree = 0.9, n_estimators = 200.

(6) Model Evaluation and Selection: Assess XGBoost performance on the test set. If XGBoost metrics outperform RF, confirm the model; otherwise, return to Step 5 for further parameter tuning or Step 2 to refine feature selection.

(7) Probability Prediction and Classification: Utilize the trained XGBoost model to output prediction probabilities:

(3) $$P\left(y=1|x\right)=\sigma \left(\sum_{k=1}^{r}f_{k}\left(x_{s*}\right)\right)$$

Here σ represents the logistic function.

Classify model output probabilities into 5 levels based on preset thresholds: Very High–Very Low, generating a landslide susceptibility map.

(8) SHAP Analysis and Visualization: Interpret factor contributions and plot the landslide susceptibility map.

(9) Retraining and Updating: Periodically supplement with new samples and InSAR monitoring results for retraining to adapt to environmental changes and enhance dynamic prediction capabilities.

## 4. SBAS-InSAR Deformation Estimation and Landslide Detection

### 4.1. InSAR Data Sources

**Figure 5:**
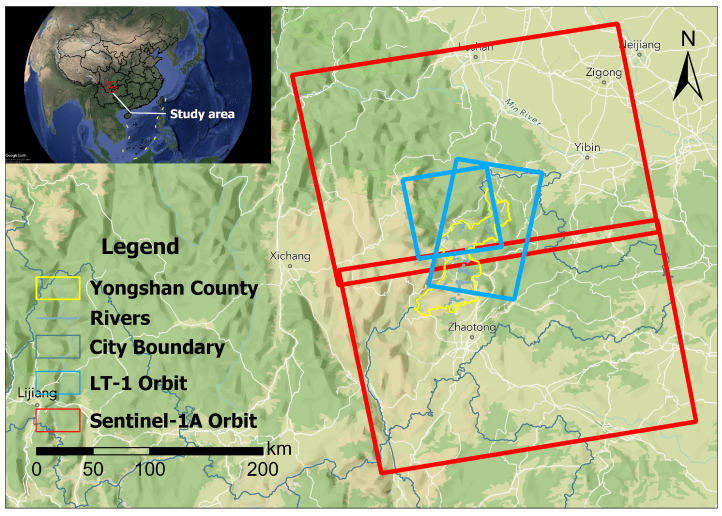
SAR data coverage in the study area showing Sentinel-1A and LT-1 satellite tracks.

This study utilizes Sentinel-1A satellite C-band SLC imagery and LT-1 satellite data, with orbital distributions shown in Figure 5.

The radar data parameters used in this study are summarized in Table 3. To enhance orbital accuracy and reduce systematic errors in radar data, precise ephemeris data from POD (Probability of Detection) were employed for orbital correction. To mitigate terrain phase effects, the Copernicus Digital Elevation Model (DEM), provided by the Geospatial Data Cloud, with a resolution of 30 m, was utilized as the external DEM.

### 4.2. SBAS-InSAR Deformation Processing and Results

Image processing in this study employed Gamma software. The temporal baseline threshold ranged from a minimum of 12 days to a maximum of 96 days, while the vertical baseline threshold spanned from a minimum of 0.4 m to a maximum of 309.2 m. The baselines exhibited good spatial connectivity. Phase detangling was performed using the Minimum Cost Flow (MCF) method with the following parameters: detangling decomposition level set to 1, correlation threshold set to 0.4, intensity threshold set to 0.02, and Goldstein filtering applied.

**Figure 6:**
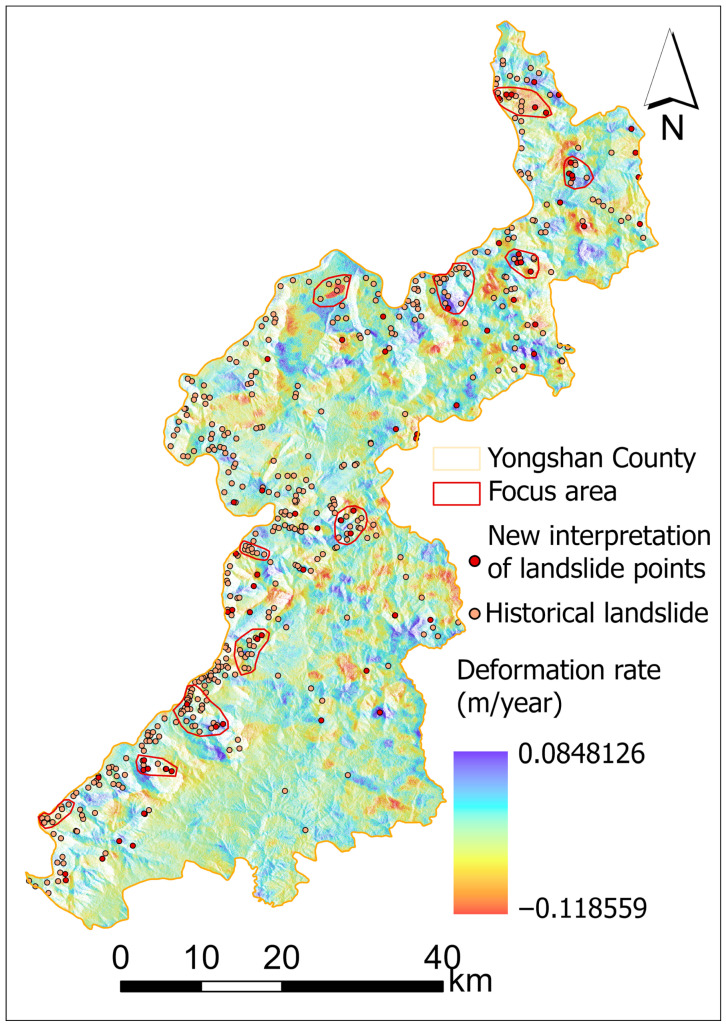
SBAS-InSAR deformation results map showing surface deformation rates and 73 identified landslide points.

SBAS-InSAR processing was applied to Sentinel-1A ascending and descending orbit data for Yongshan County from 5 May 2022, to 26 March 2025, yielding the annual average LOS-direction deformation rate for the study area, as shown in Figure 6.

Due to the absence of Sentinel-1A descent orbit coverage in Yongshan County during the period from 5 May 2022, to 26 May 2025, only based on the ascending orbit data, the annual average deformation rate in the ascending direction for Yongshan County ranges from −118.6 to −84.8 mm/year. Areas with relatively higher deformation rates farther from the ground (indicated by negative rates) are primarily located in Xiluodu Town and Qingsheng Township in northern Yongshan and Tuanjie Township in the east. Areas with relatively higher deformation rates toward the ground (positive rates) were mainly found in Daxing Town in the southwest and Lianfeng Town in the west. In summary, parts of Yongshan County exhibited high deformation rates, primarily concentrated along both banks of the Jinsha River basin. Conversely, ascent orbit data indicated low deformation rates in the county seat area and central study region, suggesting greater surface stability in these zones.

### 4.3. Interpretation of Landslide Identification Results

Landslide detection was based on the following criteria: (1) annual average deformation rate |v|>40 mm/year; (2) slope gradient $>5^{\circ }$; (3) continuous deformation for ≥6 months; (4) occurrence within geologically fragile zones (e.g., fault belts, loose deposits, cut slopes); (5) proximity to roads, rivers, or engineering activities.

Based on deformation data and Gaofen imagery, the aforementioned deformation points were overlaid onto the 2023 Gaofen imagery. The overall shape of clustered points was assessed to determine the presence of elongated, circular, elliptical, or irregular polygonal patterns to observe the presence of distinct landslide bodies, post-slide walls, and landslide boundaries. Following these identification rules and integrating deformation data with high-resolution imagery, this study identified 73 landslide or potential landslide zones.

**Figure 7:**
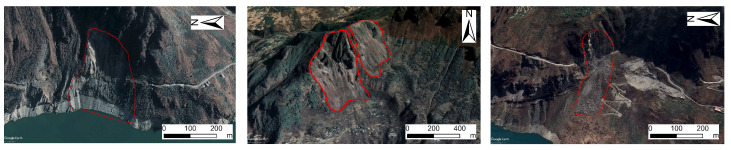
Landslide Identification Using Google Earth Historical Imagery. The red circles highlight areas of significant surface deformation and identified landslide hazards within the study region.

These landslides are primarily distributed in Daxing Town (southwest Yongshan County), Lianfeng Town (west), Xiluodu Town (north), and Qingsheng Township. Through deformation rates and optical imagery, these areas exhibit exposed bedrock and distinct landslide indicators in optical images (rock and soil fragmentation, cracks, loose structures, and sparse vegetation),as shown in Figure 7.

Field verification at selected landslide sites confirmed the high reliability of landslide interpretation using SBAS-InSAR deformation rates combined with Gaofen satellite imagery. Consequently, the aforementioned 73 landslides can be utilized for updating landslide catalogs and constructing training datasets. Additionally, LOS-oriented deformation rate results can be applied to refine landslide susceptibility mapping.

## 5. Experiments and Analysis

### 5.1. Screening of Influencing Factors

**Figure 8:**
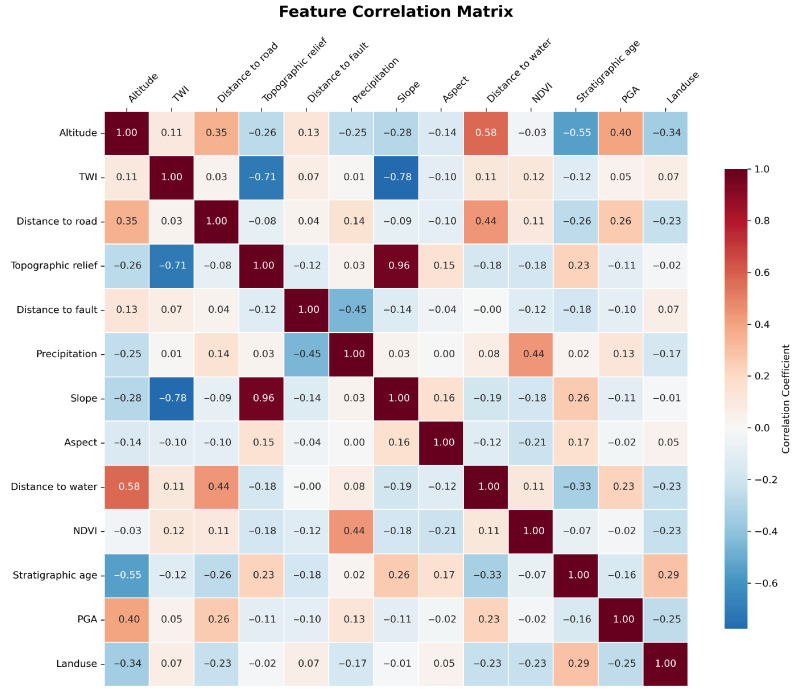
Correlation heatmap of landslide influencing factors showing relationships between different variables.

Correlation analysis was performed on the aforementioned 13 factors by calculating Pearson correlation coefficients and plotting a heatmap to represent linear correlations among evaluation factors. As shown in Figure 8, slope and relief exhibit the strongest positive correlation (r > 0.8), necessitating the elimination of one of these two factors. Other indicators fall within |r| < 0.6, indicating acceptable independence and suitability as landslide prediction factors in machine learning models. Based on the importance ranking of influencing factors, the terrain undulation factor was ultimately eliminated, retaining the remaining 12 factors for subsequent landslide susceptibility assessment.

**Figure 9:**
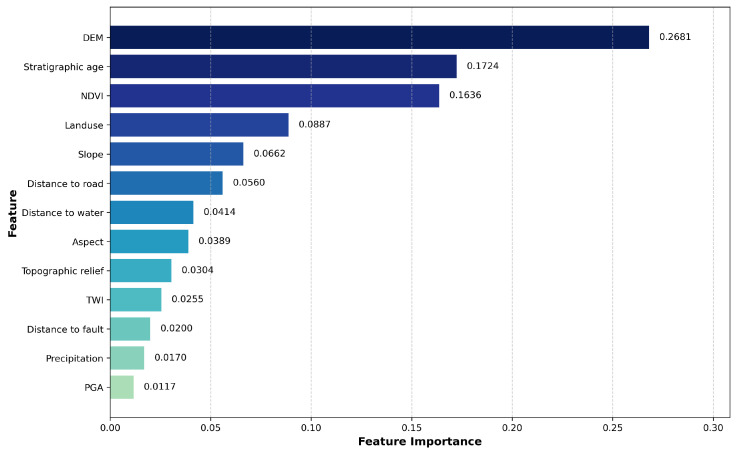
Feature importance analysis using SHAP values showing the relative contribution of each factor.

Figure 9 displays the global feature importance, where the average absolute SHAP value of each feature across all given samples is considered as its importance. Altitude, lithology, and NDVI all exhibit high importance, while distance to water and distance to road show moderate importance. This indicates that altitude, lithology, and NDVI are the core influencing factors for landslide occurrence, while human engineering activities and hydrometeorological conditions are important triggering factors.

**Figure 10:**
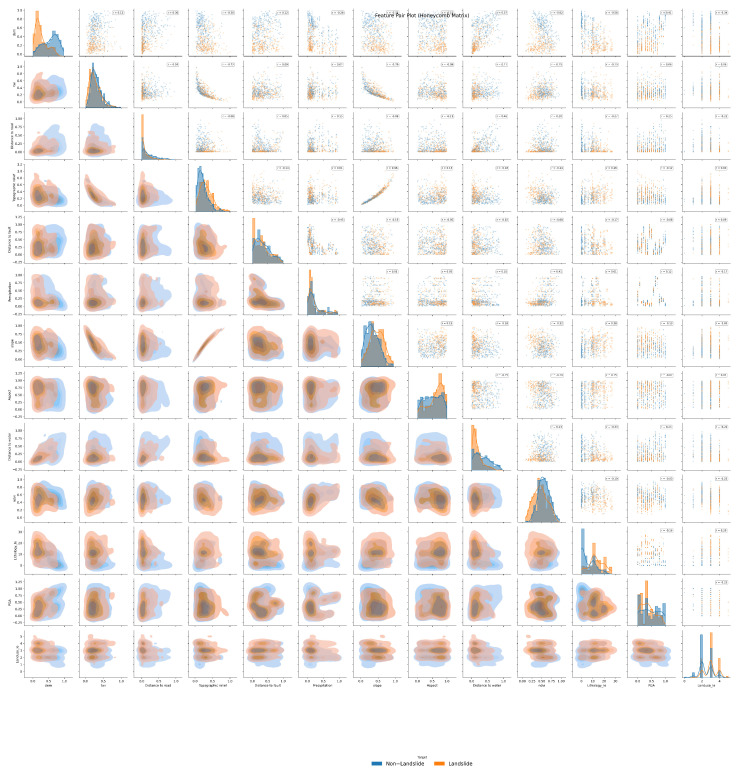
Honeycomb plot showing complex relationships among influencing factors and their joint effects on landslide occurrence.

Figure 10 reveals the complex relationships among influencing factors. Landslide points (Target = 1) cluster within specific combinations of DEM and precipitation. In the joint distribution of distance to road and slope, landslide points exhibit distinct clustering patterns. The probability of landslides significantly increases when elevation exceeds 2000 m and precipitation exceeds 1500 mm. Both proximity to roads (engineering disturbance) and excessive distance from roads (geologically vulnerable zones) increase landslide susceptibility.

### 5.2. Model Results

To effectively reduce partitioning errors and enhance the accuracy of landslide interpretation, thereby strengthening regional disaster prevention and mitigation capabilities, this study comprehensively compared the performance of multiple machine learning models in landslide susceptibility assessment. The specific models employed include standalone models RF, XGBoost, CatBoost, LightGBM, and the ensemble model RFE-RF-XGBoost.

**Figure 11:**
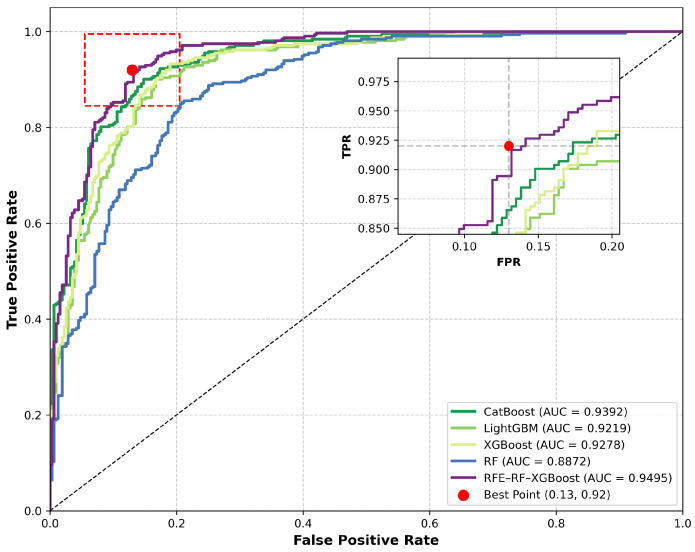
ROC curves for each model showing predictive performance comparison.

Accuracy validation of the five models revealed that all models achieved accuracy and F1 scores exceeding 0.78, with AUC values surpassing 0.88 (Figure 11).

This indicates strong fitting capability and predictive performance across all models, making them suitable for landslide susceptibility assessment in Yongshan County. The detailed performance metrics are presented in Table 4.

In terms of overall performance ranking, RFE-RF-XGBoost achieved the best results across all evaluation metrics (accuracy 0.8096, precision 0.8014, recall 0.8204, F1 score 0.8049, AUC = 0.9495), significantly outperforming other models. It particularly excelled in identifying high-risk zones, making it suitable for disaster prevention and mitigation applications requiring extremely high accuracy. The CatBoost model ranked second overall (AUC = 0.9392) with high recall, indicating strong recognition capability for actual landslide samples and suitability for landslide risk early warning scenarios. XGBoost and LightGBM models demonstrated comparable performance, both exhibiting solid comprehensive capabilities and smooth prediction outputs, making them suitable for preliminary large-scale susceptibility zoning. Although the RF model had a relatively lower AUC value (0.8872), it still provided fundamentally usable prediction performance and can serve as a baseline model or for comparative validation.

In summary, the RFE-RF-XGBoost model demonstrates significant advantages in improving zoning accuracy and enhancing the identification of extremely high-risk zones, making it the preferred model for high-precision landslide susceptibility assessment. For different application scenarios, other models such as CatBoost or XGBoost can be flexibly selected based on varying priorities for metrics like precision and recall.

### 5.3. Partitioning Ratios and Field Validation

**Figure 12:**
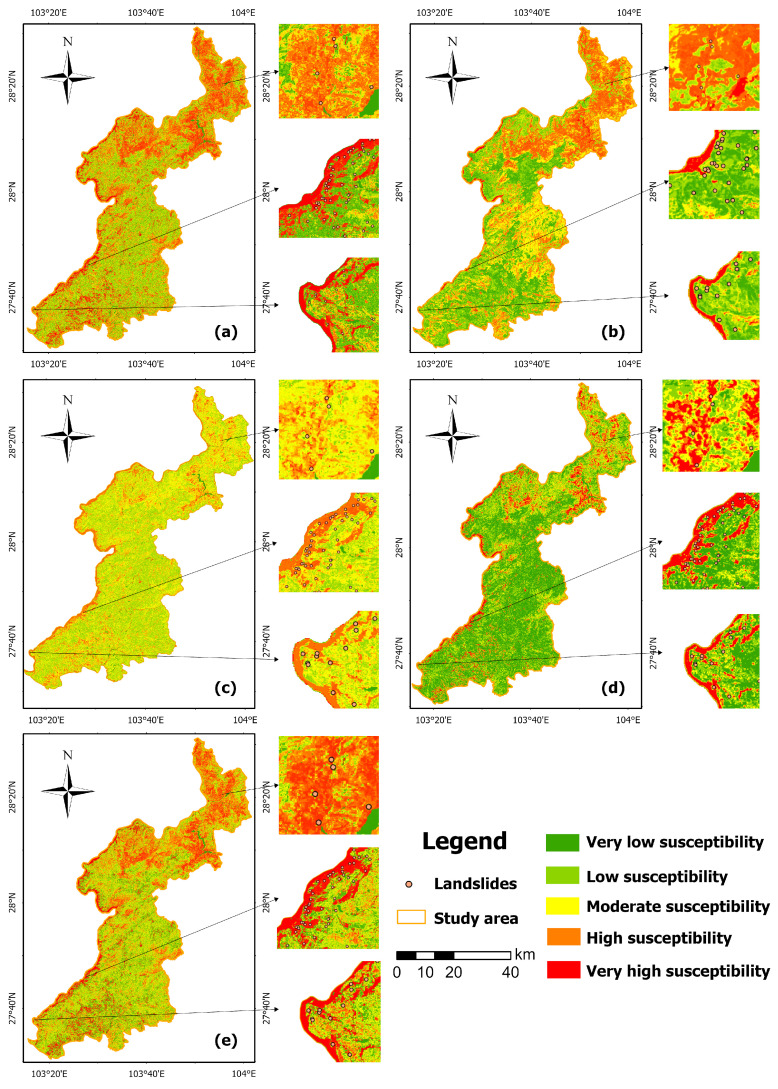
Landslide susceptibility mapping results from five different models: (**a**) RF, (**b**) CatBoost, (**c**) LightGBM, (**d**) XGBoost, (**e**) RFE-RF-XGBoost.

Machine learning-based landslide susceptibility assessment employed the Scikit-learn library in Python 3.13.2, training five combined models: CatBoost, LightGBM, XGBoost, RF, and RFE-RF-XGBoost.The prediction outcomes were incorporated into ArcGIS and subsequently classified through the natural breaks (Jenks) method, with the resultant susceptibility zonation presented in Figure 12.

The spatial distributions of landslide susceptibility assessments across models exhibit high consistency. High and extremely high susceptibility zones are concentrated in western and northeastern Yongshan County, primarily along riverbanks. These areas provide favorable conditions for landslide initiation due to their downstream river locations, frequent human activity, steep topography, and active fault zones. Conversely, extremely low to low susceptibility zones are predominantly distributed in central and southeastern Yongshan County. These sparsely populated areas are distant from water bodies, resulting in better soil conditions and stronger shear resistance, which are unfavorable for landslide development.

As shown in Figure 12, the number of landslide points is directly proportional to the landslide susceptibility rating: higher susceptibility ratings correspond to a greater number of landslide points.

Using the attribute matrix of the entire grid points as model input, the corresponding susceptibility indices for each model were obtained and classified into five levels based on the following criteria: Extremely Low Susceptibility (P(S)<0.05), Low Susceptibility (0.05≤P(S)<0.15), Moderate Susceptibility (0.15≤P(S)<0.40), High Susceptibility (0.40≤P(S)<0.70), and Very High Susceptibility (P(S)≥0.70).

The susceptibility distributions for each model are shown in Figure 12. The high-risk landslide areas predicted by the RF, CatBoost, and RFE-RF-XGBoost models are primarily distributed along the Jinsha River and in the eastern and northern regions, exhibiting a relatively uniform and reasonable overall distribution. In contrast, the LightGBM model predominantly predicts low- and medium-risk areas, with a smaller proportion of high- and very high-risk zones. The XGBoost model results show smooth transitions between risk levels, resulting in a relatively continuous distribution.

**Figure 13:**
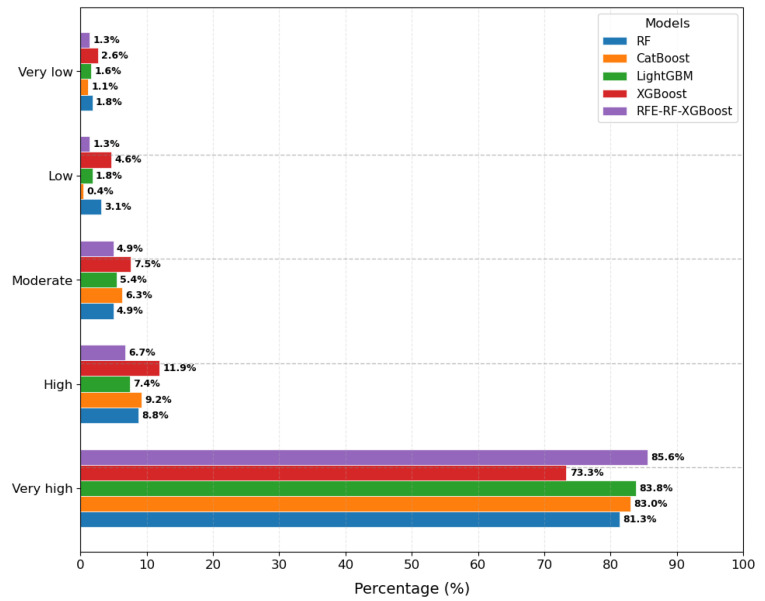
Percentage distribution of landslide units across susceptibility grades for each model.

The percentage distribution of landslide units across susceptibility grades for each model is shown in Figure 13. Results indicate that all five machine learning models demonstrate effective zoning capabilities. Among them, the RFE-RF-XGBoost model exhibits optimal mapping performance, achieving an 85.6% identification rate in the ’Very High’ risk category while maintaining better error control in the ’Low’ and ’Moderate’ risk classifications. This model delivers the most stable overall performance.

**Figure 14:**
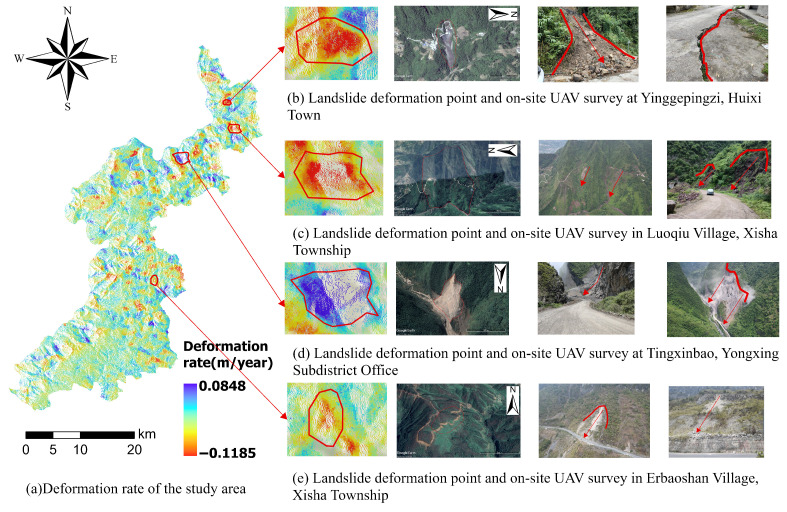
Field verification of landslides: four case studies validating the susceptibility mapping results. The red arrows indicate the direction of landslide movement or the potential sliding trend.

The applicability of landslide susceptibility mapping is further analyzed through four specific case studies shown in Figure 14.

1. Yinggepingzi landslide (Huixi Town): The landslide exhibits a tongue-shaped plan view, a concave profile, and a linear slip surface. The overall slope angle is approximately 40°, with a primary slip direction of 85°. Field investigation revealed subsidence cracks on the rear wall road, with buildings tilting due to ground collapse. Figure 14 indicates the RFE-RF-XGBoost model predicts high-to-extremely high susceptibility for this landslide.
2. Luoqiu Village landslide (Xisha Township): This landslide area features intensely dissected terrain with steep gradients primarily influenced by human road construction and slope-cutting activities. The landslide structure is a composite rock-soil slope with a longitudinal orientation, posing a potential risk of rockfall hazards. The model predicts high-to-extremely high susceptibility for this landslide.
3. Tingxinbao landslide (Yongxing Subdistrict Office): Located on the left bank of the Fotan River, field investigations indicate it is a collapse or landslide hazard site. Construction of this quarry commenced in March 2012, and stone extraction is currently underway. The RFE-RF-XGBoost model predicts high susceptibility for this landslide.
4. Erbaoshan landslide (Xisha Township): Located on a steep mountain slope with cliffs, the slope exhibits a terraced morphology with significant topographic variations. The landslide susceptibility mapping indicates this landslide as highly susceptible according to the RFE-RF-XGBoost model prediction.

Validation through four typical landslides—Yinggepingzi, Luoqiu Village, Tingxinbao, and Erbaoshan—shows that the model accurately identifies high-risk areas of landslides with diverse triggers and morphological characteristics. Whether for composite rock–soil slopes disturbed by road excavation or potential collapse bodies on steep terrain, the model successfully predicted these as “high-to-extremely high” susceptibility zones. These predictions align closely with field observations of instability signs, such as subsidence cracks, building tilting, and rock mass loosening, confirming the model’s precision in complex real-world scenarios. Furthermore, these findings provide a scientific basis for local governments to update regional landslide inventory maps and implement protective measures—such as constructing retaining walls and passive protection nets in high-susceptibility areas—thereby enhancing regional disaster prevention and mitigation capabilities.

### 5.4. Summary

Using Yongshan County, Yunnan Province as a case study, this paper employs Sentinel-1A satellite ascending orbit data and high-resolution remote sensing imagery to identify landslide areas. Five machine learning models were applied for landslide susceptibility assessment, yielding zoned evaluation results. These were then refined through susceptibility evaluation and adjusted using deformation rate data, leading to the following conclusions:

1. Key influencing factors: The main influencing factors in Yongshan County include Elevation, Slope, Aspect, Land Use, TWI, Rainfall, NDVI, Distance to Roads, Distance to Rivers, Distance to Faults, PGA, and Stratigraphic Lithology. This aligns with the county’s actual conditions, where most landslides are triggered by fragile geological conditions, seasonal rainfall, earthquakes, urban residential development, and large-scale reservoir impoundment.
2. SBAS-InSAR monitoring results: SBAS-InSAR technology was employed to monitor deformation data from 5 May 2022 to 26 March 2025. Results indicate an ascending orbit LOS deformation rate of −118.6 to −84.8 mm/year, with high-deformation zones primarily located in Xiluodu Town and Qingsheng Township in northern Yongshan, and Tuanjie Township in the east. Integration of high-resolution remote sensing imagery with landslide susceptibility results identified 73 landslide-prone areas and updated the landslide dataset.
3. Model performance: The ROC accuracy of the RFE-RF-XGBoost algorithm showed significant improvement over single models, outperforming RF by 6.2%, XGBoost by 2.2%, CatBoost by 1%, and LightGBM by 2.8%. The model identified 85.6% of landslides in extremely high susceptibility zones, while only 1.3% occurred in low-susceptibility zones.
4. Deformation zone identification: The SBAS-InSAR derived deformation map revealed several active deformation zones, most within known landslide boundaries (e.g., the middle to front edge of Sifangbei landslide). It also identified previously undelineated active zones, such as around the Dbaishu landslide, which may develop into larger-scale landslides.
5. Integrated susceptibility mapping: A comprehensive landslide susceptibility map was constructed by integrating static susceptibility from the RFE-RF-XGBoost model with dynamic deformation velocity from InSAR. This approach captures both spatial characteristics from static factors and temporal evolution features from dynamic deformation, enhancing assessment reliability.

## 6. Discussion

### 6.1. Model Performance and Factor Contribution Analysis

The results of this study demonstrate that the RFE-RF-XGBoost model achieved the best performance in landslide susceptibility assessment in Yongshan County. SHAP analysis revealed that elevation, stratigraphic lithology, and NDVI are the core influencing factors, which aligns with the local geological environment characterized by high mountains, deep valleys, and drastic vegetation changes. The importance of distance to roads and distance to rivers highlights the significant triggering roles of human engineering activities and river erosion.

### 6.2. Comparison with Existing Studies

Our findings are consistent with studies by Huang et al. [4] and Liu et al. [14], confirming that ensemble models generally outperform single models. However, this study further mitigates overfitting risks with high-dimensional data by introducing RFE for feature pre-screening, representing an advancement over traditional ensemble methods such as simple bagging or boosting.

### 6.3. Advantages and Limitations of the Innovative Method

Integrating SBAS-InSAR deformation data directly as input features for the machine learning model, compared to the matrix overlay method used by Ciampalini et al. [21], more directly captures dynamic information of slope movement, enabling a combined assessment in both temporal and spatial dimensions. However, this method also faces challenges: firstly, InSAR signal decorrelation in densely vegetated or steep terrain leads to data gaps; secondly, the model construction and hyperparameter tuning incur high computational costs, suggesting the potential for exploring more efficient automated machine learning pipelines in the future.

### 6.4. Implications for Disaster Risk Management

The high-resolution susceptibility maps produced by this study provide direct scientific support for territorial spatial planning and disaster risk prevention and control in Yongshan County. Identified ’very high-risk’ areas should be prioritized for deploying monitoring equipment and implementing engineering mitigation measures.

### 6.5. Parameter Tuning Strategy and Computational Costs

The grid search method adopted in this study, while effective in identifying near-optimal hyperparameter combinations, incurs computational costs that grow exponentially with parameter dimensionality. The complete model training process, executed on a workstation equipped with an Intel i7 processor and 64GB RAM, required approximately 1.5 h to finalize. For future large-scale applications, more efficient hyperparameter optimization strategies such as Bayesian optimization or randomized search could be implemented to better balance computational efficiency with predictive performance.

## 7. Conclusions

This study integrates SBAS-InSAR technology with machine learning methods. By utilizing SBAS-InSAR to obtain high-precision surface deformation rates as key dynamic indicators for machine learning models, it effectively improves the accuracy of landslide susceptibility zoning and reduces zoning errors. The following conclusions are drawn:

- By integrating InSAR deformation rates with optical remote sensing imagery for landslide hazard identification, a total of 73 landslide or potential landslide zones were detected. The interpreted landslides exhibited slopes ranging from 10° to 45° and posed clear threats to nearby objects. Hazard points were primarily located along both banks of the Jinsha River basin, as well as in Daxing Town (southwest Yongshan County), Lianfeng Town (west Yongshan County), Xiluodu Town (north Yongshan County), and Qingsheng Township (north Yongshan County).
- Building upon this, an RFE-RF-XGBoost model incorporating boundary conditions was developed to reduce zoning errors. This model demonstrated optimal mapping performance, reducing the proportion of low-risk zones by 2–4% while increasing the proportion of extremely high-risk zones by approximately 2–12%. The model achieved an AUC value of approximately 0.95, indicating strong generalizability.

This study holds promising applications for landslide identification and risk prevention, achieving accurate detection of potential landslide areas while providing timely and reliable analytical tools for geological hazard risk management. However, several limitations warrant further exploration and improvement during technical implementation, specifically in the following two aspects:

- InSAR technology is susceptible to signal instability in areas with dense vegetation or steep topography. Introducing complementary multi-source data such as L-band satellite imagery, optical remote sensing, and LiDAR can enhance monitoring accuracy and reliability;
- While landslide susceptibility assessment reveals spatial probabilities, truly serving disaster prevention and mitigation requires incorporating exposure factors such as buildings, roads, and population density to transition toward risk assessment. This comprehensive approach evaluates potential losses and supports emergency decision-making.

## Figures and Tables

**Table 1:** Data sources and description.

Indicator Factor	Source	Data Description
GF-2 satellite imagery	https://www.ovital.com/ (accessed on 20 November 2025)	0.8 m spatial resolution
Google Earth imagery	https://www.google.cn/ (accessed on 20 November 2025)	0.3–0.6 m spatial resolution
DEM	https://www.gscloud.cn/ (accessed on 20 November 2025)	GDEM V3 30 m resolution digital elevation data
Landslides	https://ynddj.org.cn/ (accessed on 20 November 2025)	Detailed landslide survey/inventory of Yongshan County
Geological age	https://www.ngac.cn/ (accessed on 20 November 2025)	1:200,000 geological map
Land-use types	https://livingatlas.arcgis.com (accessed on 20 November 2025)	Vector data of land use type in 2023
Hydrological network & roads	https://openstreetmap.org/ (accessed on 20 November 2025)	2023 vector data of rivers and roads
Faults	https://www.activefault-datacenter.cn (accessed on 20 November 2025)	1:50,000 strip map of the North–South Seismic Belt
NDVI	Google Earth Engine	2023 30 m resolution NDVI
Rainfall	Google Earth Engine	2023 mean annual rainfall, 5 km resolution
Peak ground acceleration	https://www.gb18306.net/ (accessed on 20 November 2025)	China Seismic Ground-motion Parameters Zonation Map

**Table 2:** XGBoost Hyperparameter Tuning Range.

Hyperparameter	Candidate Values
learning_rate	[0.01, 0.05, 0.1, 0.2]
max_depth	[3, 5, 7, 9]
min_child_weight	[1, 3, 5]
gamma	[0, 0.1, 0.2]
subsample	[0.8, 0.9, 1.0]
colsample_bytree	[0.8, 0.9, 1.0]
n_estimators	[100, 200, 300]

**Table 3:** Radar data source parameters for Sentinel-1A and LT-1 satellites.

Parameter	Sentinel-1A	LT-1
Temporal coverage	May 2022–Mar 2025	Jan 2024–Feb 2025
Frame Start-Frame End	128-84 and 128-89	341,494 and 300,332
Repeat cycle	12 days	27 days
Polarization	VV + VH	HH
Operating Mode	IW	-
Swath Width	180 km	55 km

**Table 4:** Performance comparison of different machine learning models for landslide susceptibility assessment.

Model	Accuracy	Precision	Recall	F1-Score	AUC
CatBoost	0.7903	0.7811	0.8045	0.7925	0.9392
LightGBM	0.7791	0.7721	0.7894	0.7806	0.9219
XGBoost	0.7865	0.7819	0.7889	0.7849	0.9278
RF	0.7791	0.7681	0.7971	0.7823	0.8872
RFE-RF-XGBoost	0.8096	0.8014	0.8204	0.8049	0.9495

## Data Availability

The data presented in this study are available on request from the corresponding author. The data are not publicly available due to privacy restrictions.

## Abbreviations

To enhance readability for a broader audience, the following table provides the full names and brief explanations of the technical acronyms used throughout this paper.

Acronym	Full Name
SBAS-InSAR	Small BAseline Subset Interferometric Synthetic Aperture Radar
LT-1	LuTan-1 (A Chinese L-band SAR satellite)
RFE	Recursive Feature Elimination
DEM	Digital Elevation Model
RF	Random Forest
LOS	Line-Of-Sight
XGBoost	eXtreme Gradient Boosting
NDVI	Normalized Difference Vegetation Index
CatBoost	Categorical Boosting
TWI	Topographic Wetness Index
LightGBM	Light Gradient Boosting Machine
PGA	Peak Ground Acceleration
SAR	Synthetic Aperture Radar
ROC	Receiver Operating Characteristic
SLC	Single Look Complex
AUC	Area Under the Curve
PS-InSAR	Persistent Scatterer InSAR
SHAP	SHapley Additive exPlanations
InSAR	Interferometric Synthetic Aperture Radar
SMOTE	Synthetic Minority Over-sampling Technique
GIS	Geographic Information System
CV	Cross-Validation
